# Inhibition of ROCK1 kinase modulates both tumor cells and stromal fibroblasts in pancreatic cancer

**DOI:** 10.1371/journal.pone.0183871

**Published:** 2017-08-25

**Authors:** Clifford J. Whatcott, Serina Ng, Michael T. Barrett, Galen Hostetter, Daniel D. Von Hoff, Haiyong Han

**Affiliations:** 1 Molecular Medicine Division, The Translational Genomics Research Institute, Phoenix, Arizona, United States of America; 2 Mayo Clinic Cancer Center, Scottsdale, Arizona, United States of America; 3 Laboratory of Analytical Pathology, The Van Andel Research Institute, Grand Rapids, MI, United States of America; University of South Alabama Mitchell Cancer Institute, UNITED STATES

## Abstract

ROCK, or Rho-associated coiled coil-containing protein kinase, is a member of the AGC kinase family and has been shown to play a role in cell migration, ECM synthesis, stress-fiber assembly, and cell contraction. Increased ROCK expression has been reported in multiple pathological conditions, including cancer. Here, we report increased expression of ROCK 1 in pancreatic tumor epithelial cells as well as in cancer associated fibroblasts (CAF). In our analysis, 62% of tumor samples exhibited ≥2+ in staining intensity by IHC analysis, versus 40% of adjacent normal tissue samples (P<0.0001). Thus, we hypothesized that ROCKs may play a significant role in pancreatic cancer progression, and may serve as a suitable target for treatment. We report a low frequency (4/34) amplification of the ROCK1 gene locus at chromosome 18q11.1 in pancreatic ductal adenocarcinoma (PDAC) patient tissue samples by aCGH analysis. Inhibition of ROCK kinase activity by a small molecule inhibitor (fasudil) resulted in moderate (IC_50_s of 6–71 μM) inhibition of PDAC cell proliferation, migration, and activation of co-cultured stellate cells. In the KPC mouse model for pancreatic cancer, fasudil decreased tumor collagen deposition. This translated to an enhanced overall survival of the mice and an increase in gemcitabine uptake. Though fasudil may target both the tumor epithelial cells and the CAFs, our findings are consistent with the hypothesis that inhibition of tumor stroma enhances drug penetration and efficacy in PDAC. Overall, our data suggests that ROCK1 may serve as a potential therapeutic target to enhance current treatment regimens for pancreatic cancer.

## Introduction

RhoA/ROCK1 signaling has been shown to play an important role in cancer development and progression [1,2]. RhoA acts downstream of various G protein-coupled receptors, and can be induced by TGFβ ligand binding [3,4]. ROCK1 is the key mediator of RhoA activity, and is a multifunctional member of the AGC (protein kinase A/G/C) kinase family that has also been implicated in the modulation of stress fiber assembly, cell contraction, apoptosis, migration, and invasion of multiple cancer cell types. ROCK1 mediates the Smad-independent, TGFβ/RhoA signaling axis, and has also been shown to be an important mediator of cancer-associated fibroblast (CAF) activation and deposition of extracellular matrix (ECM) proteins in solid tumors [5,6]. ROCK1 substrates include LIM kinase (LIMK), Myosin light chain (MLC), and Myosin phosphatase target subunit 1 (MYPT1) [1]. Inhibition of ROCK activity in tumor cells decreases migration and invasive capacity in pancreatic cancer [7,8]. The activity of RhoA/ROCK1 is of particular interest; however, because of its role in dysregulated ECM deposition in CAFs, a characteristic component of the clinico-pathologic phenomenon termed desmoplasia.

The macroscopic changes described in desmoplasia arise in large part from extensive proliferation of myofibroblast cells that, upon activation, secrete extracellular matrix proteins that accumulate in the stromal compartment of solid tumors [9]. This response, though common in the wound healing process, is not resolved in tumor tissue, leading to high levels of accumulating ECMs [10]. Multiple signaling pathways and multiple cell types have been identified as central to the desmoplastic response in cancer. Important components include: 1) platelet-derived growth factor (PDGF) signaling as central to myofibroblast cell proliferation, 2) transforming growth factor β (TGFβ), central to initiating myofibroblast activation, and 3) deposition of ECM proteins. Deposition of ECM proteins can contribute to poor tumor perfusion and diffusion of drugs [11]. Pathophysiologic components and clinical patient response have shown that desmoplasia is a highly relevant feature to the survival of patients suffering from pancreatic cancer [12].

Pancreatic ductal adenocarcinoma (PDAC) mortality remains significant, with a 5-year survival rate of around 8% in the United States [13]. Both molecular and physiological chemoresistance in pancreatic tumors contribute to this poor patient survival. The gemcitabine plus nab-paclitaxel combination and the FOLFIRINOX regimen are the current first-line therapies for patients with advanced pancreatic cancer [14,15]. The gemcitabine plus nab-paclitaxel combination treatment offers a median survival in patients with advanced disease of 8.5 months [15], whereas the median survival of FOLFIRINOX treated patients is 11.1 months [14]. While improvements to patient survival have been made in such advances, the majority of patients will progress after 6 months of treatment. New therapies with greater efficacy are urgently needed for this disease. To this end, targeting tumor desmoplasia to improve drug delivery and overcome chemoresistance is being investigated as a new therapeutic approach.

We hypothesize that ROCK1 targeting may enable such a therapeutic approach. Activating mutations have been identified in ROCK1 in some cancer types [16]. These mutations result in a more aggressive and migratory phenotype in these tumors. Altered ROCK1 expression has been shown in breast tumors, osteosarcoma, and pancreatic cancer [7,17,18]. In this study we further explore the role of ROCK1 in the desmoplasia, chemoresistance, and progression of PDAC and its potential as a therapeutic target.

## Materials and methods

### Materials

Gemcitabine and fasudil were purchased from LC Laboratories (Woburn, MA, USA). Anti-ROCK1 antibodies (C-19) were purchased from Santa Cruz Biotechnology (Dallas, TX, USA). Collagen I, α-SMA, and CD31 antibodies were purchased from Abcam (Cambridge, MA, USA). ROCK1 siRNAs were obtained from QIAGEN (Valencia, CA, USA). All other reagents, including desmin antibodies, were purchased from Sigma-Aldrich (St. Louis, MO, USA), unless otherwise noted.

Pancreatic cancer cell lines, PANC-1, SU.86.86, BxPC3, AsPC-1, HS766T, and Mia PaCa-2 were purchased from American Type Culture Collection (ATCC) and cultured in RPMI-1640 media supplemented with 10% FBS. HPDE6 was kindly provided by Dr. Ming-sound Tsao at Princess Margaret Cancer Centre and was cultured in keratinocyte media (Invitrogen, now part of ThermoFisher Scientific, Carlsbad, CA). All cell lines were authenticated by STR profiling using the AmpFISTR Identifiler PCR amplification kit (Applied Biosystems, Foster City, CA). The STR profiling results were compared to published STR sequences from the ATCC or their original sources to ensure authenticity. The STR profiling was repeated once a cell line had been passaged more than 6 months after previous STR profiling. The CW1 fibroblast cells were isolated following protocols described elsewhere from a resected PDAC patient’s tumor under an Institutional Review Board (IRB) approved protocol [19,20].

### Human PDAC tissues

All human tissues included in study were collected as part of an NIH P01 program project grant (CA109552) where patient samples were collected under protocols approved by the Western Institutional Review Board (WIRB). Informed consent was obtained from patients included in study. Diagnostic and histomorphologic confirmation to include percentage tumor and stromal cellularity were performed by a board-certified pathologist (GH).

### aCGH analysis

Patient samples were flow sorted prior to genomic analysis using our published DNA content based protocols [21]. Briefly, DNAs were extracted using QIAGEN micro kits (Valencia, CA, USA). For each hybridization, 100 ng of genomic DNA from each sample and of pooled commercial reference (Promega, Madison, WI, USA) were amplified using the GenomiPhi amplification kit (GE Healthcare, Piscataway, NJ, USA). Subsequently, 1 ug of amplified sample and 1 ug of amplified reference template were digested with DNasel then labeled with Cy-5 dUTP and Cy-3 dUTP, respectively, using a BioPrime labeling kit (Invitrogen, Carlsbad, CA, USA). All labeling reactions were assessed using a Nanodrop assay (Nanodrop, Wilmington, DE, USA) prior to mixing and hybridization to CGH arrays with either 244,000 or 400,000 oligonucleotide features (Agilent Technologies, Santa Clara, CA, USA). The aCGH data have been deposited in the National Center for Biotechnology Information (NCBI) Gene Expression Omnibus (accession numbers GSE54328 and GSE21660).

### Immunohistochemical staining and assessment

Tumor regions were selected by the study pathologist (GH) for inclusion in this study. Two to three distinct regions of each formalin-fixed paraffin-embedded (FFPE) tumor block, and when available adjacent normal pancreas, were sampled using 1 mm diameter cores in the construction of our tissue microarray (TMA). TMA construction was performed using a Veridiam Semi-Automated Tissue Arrayer (VTA-100).

TMA blocks were sectioned at 5μm and stained using procedures as described previously [22]. Briefly, following attachment to the slide, sections were deparaffinized with xylenes, rehydrated through graded ethanol baths and antigen retrieved using a BondMaxTM autostainer (Leica Microsystems, Bannockburn, IL). Slides were developed using the BondTM Polymer Refine Detection kit (Leica) using 3,3’-diaminobenzidine tetrahydrochloride chromogen as substrate. ROCK1 antibody (Santa Cruz Biotechnology) was used at 1:125 dilution. Staining intensity was assessed upon review of multiple random snapshots taken from digitally scanned slides. ROCK1 staining intensity was assessed on a scale of 0–3+, where 0 is negative, 1+ is weak staining, 2+ is moderate staining, and 3+ is strong staining.

Immunostaining of mouse tumor sections was also carried out using the BondMaxTM autostainer and the BondTM Polymer Refine Detection kit. Antigen retrieval was performed using a heat-induced antigen retrieval protocol with a citrate based epitope retrieval solution (pH 6.0, Leica Microsystems). The dilution and incubation time for the primary antibodies used were: Ki67 (Abcam, Cat# ab16667), 1:100 dilution for 30 minutes; Cleaved Caspase 3 (CC3, Biocare Medical, Cat# CP229A), 1:40 for 30 minutes; Desmin (Sigma Aldrich, Cat# HPA018803), 1:250 for 30 minutes; α-SMA (Abcam, Cat# ab5694), 1:150 for 30 minutes; CD31 (Abcam, Cat# ab28364), 1:200 for 30 minutes; and Collagen I (Novus Biologicals, Cat# NB600-408), 1:50 for 30 minutes. The Movat’s pentachrome staining was carried out using a kit from American MasterTech (Cat# KTRMP) according to manufacturer’s recommended protocols.

To quantify the changes in Ki67, CC3 and CD31 positive cells, five random, 20X magnified snapshots were captured of each stained section. Images were then processed in Photoshop (Adobe) for extraction of the yellow channel (CMYK) into a grayscale image as has been described elsewhere [23]. Thresholded images were then analyzed in ImageJ for the presence of positive cells. Pentachrome analysis was peformed similarly using the yellow channel, followed by subtraction of the cyan channel to remove the contribution of mucin (green) staining to the yellow pixel count. To assess changes in metastatic lesions with treatment, resected and fixed median liver lobes were cut into three portions, placed on cut ends, and embedded in paraffin before being sectioned. Five sections were cut at periodic depths to assess a random sampling of tumor burden in the liver. The presence of metastatic lesions was assessed on H&E stained sections, by a staff pathologist.

### siRNA silencing of ROCK1 expression

ROCK1 knockdown was achieved using two siRNA duplexes, targeting distinct regions of ROCK1 transcript. ROCK1, non-targeting, and a positive, transfection control (all-star death, ASD) siRNA were delivered at 20nM using siLentFect (Bio-Rad, Hercules, CA, USA) reagent according to manufacturer protocol. Cellular viability was assessed following siRNA treatment using the sulforhodamine B (SRB) assay, as has been described previously [24,25]. Briefly, cells were first fixed with trichloroacetic acid, washed, and then stained with 0.4% SRB dye. Cells were then washed with acetic acid, dried, and then solubilized in 50mM Tris buffer. Absorbance was then assessed at 570nm.

### Immunoblotting

Cell lysates were interrogated for ROCK1 expression by SDS-PAGE and standard Western blotting techniques, with a few modifications to improve detection as has been described previously [26]. Briefly, cells were harvested and lysed in RIPA buffer (Cell Signaling Technology, Danvers, MA) containing 1x protease and phosphatase inhibitor cocktail (Roche Diagnostics Corporation, Indianapolis, IN) and incubated on ice for 30 minutes. The lysates were then centrifuged at 14,000 g for 10 minutes at 4°C to remove cell debris. Protein concentration was determined using the bicinchoninic acid (BCA) protein assay (Pierce Biotechnology, Rockford, IL). Twenty micrograms of protein per lane was separated by 4–12% NuPAGE Bis-Tris gel electrophoresis (Invitrogen). Proteins were then transferred onto PVDF membranes. Low concentrations (0.025%) of tween 20 were used in subsequent blot detection and washing steps to enhance staining detection. Finally, blots were imaged using chemiluminescence and a UVP Biospectrum imager (UVP, Upland, CA, USA). Dot blots were run using the Bio-Dot microfiltration apparatus (Bio-rad). Lysates were collected in protease/phosphatase-inhibitor containing PBS, sonicated for 5s, and filtered onto a nitrocellulose membrane under vacuum. For assessment of total protein loading, blots were stained with ponceau S and imaged. Blots were subsequently washed, and then detected using standard immunoblotting technique and antibody dilutions.

### Immunofluorescent staining

To assess α-SMA and collagen in fasudil treated cells, we used standard immunostaining technique. Briefly, following treatment, cells were washed in PBS, and then fixed in 5% formaldehyde for 30 minutes. Cells were subsequently washed in PBS, deactivated in 100mM glycine, and then washed again before being permeabilized in 0.4% Triton X-100. Cells were blocked in 3% BSA, before being detected with anti-α-SMA or collagen antibodies. Following detection and washing steps, cells were counterstained with Vectashield (Vector Laboratories) containing 1 μg/mL DAPI (Sigma).

### Migration assay

To assess the migratory capacity of Panc-1 cells, we performed a Boyden Chamber migration assay, as has been described previously [27]. Briefly, fibronectin coated chambers were placed in 10% FBS containing media (bottom well), as the chemoattractant, while 2.5x10^5^ non-targeting siRNA or ROCK1 siRNA-treated Panc-1 cells were suspended in serum-free media, and seeded in upper chambers. Cells were allowed to migrate for 24 hours before membranes were processed and harvested. Membranes were fixed in methanol and ethanol, washed and stained, then permanently mounted. Multiple images of each replicate were counted.

### Mice

All animal studies were performed in strict accordance with the recommendations in the Guide for the Care and Use of Laboratory Animals of the National Institutes of Health. The protocols were approved by the Institutional Animal Care and Use Committee (IACUC) at the Translational Drug Development Inc. (TD2) and the University of Arizona. KPC (LSL-Kras^G12D/+^; LSL-Trp53^R172H/+^; Pdx-1-Cre) mice used in the study were generated as previously described by Hingorani and colleagues [28]. Mice were housed at room temperature (70–76°F), and were given water and chow *ad libitum*. Following weaning and confirmation of their genotype, biweekly abdominal imaging of the KPC mice was performed using three-dimensional high resolution ultrasonography with Visualsonics Vevo 770 system (Fujifilm Visualsonics, Ontario, Canada). Overall, a total of 108 KPC mice were enrolled into the studies, of which 7 were found dead overnight and 101 were euthanized at the end of studies.

### KPC mouse tumor volume studies

For tumor volume studies, mice were imaged biweekly with ultrasonography and triaged according to the development of pancreatic pathology, including edema, dilation of pancreatic ducts, or the development of small tumors. Mice were imaged until the development of focal pancreatic tumors measuring at least 100mm^3^ in total volume. Once this enrollment criteria was met, mice were randomized into one of three treatment groups: vehicle (saline, 0.9% NaCl, PO, BID), gemcitabine (80mg/kg, IP, q3dx4, starting on Day 3), or gemcitabine plus fasudil (100mg/kg, PO, BID, starting on Day 1). Following each 12-day treatment cycle, mice were again imaged using ultrasonography and tumor volumes were measured post-treatment. Subsequent tumor volume measurements were normalized to the baseline, where baseline was defined as the first measurement where tumor volume exceeded 100 mm^3^, and presented as a percent change.

### KPC mouse survival studies

For mouse survival studies, KPC mice were monitored by three-dimensional high resolution ultrasonography biweekly until the development of large, focal pancreatic tumors, measuring at least 100mm^3^ in total volume. Once this enrollment criterion was met, KPC mice were randomized into one of three arms: vehicle (saline, 0.9% NaCl), gemcitabine alone (80mg/kg, IP, q3dx4), or gemcitabine plus fasudil (100mg/kg, PO, BID). Once enrolled, mice were treated with up to three, 12-day cycles (with 7-day treatment holiday between cycles), until survival endpoint criteria were met. When any two of the following endpoint criteria were met, mice were considered moribund and were euthanized immediately using: greater than or equal to 20% body weight loss, severe lethargy, significant accumulation of ascites, and significant and sustained decrease in body temperature.

### Intratumor gemcitabine concentration assessment

Intratumor gemcitabine concentrations were assessed as described previously [29,30]. Tissue samples were cut from frozen tumor samples, upon which a 50:50 mixture of acetonitrile:water containing tetrahydrouridine (THU) was added. Samples were homogenized with a tissue homogenizer (Brinkmann) for 45 seconds. To homogenates, ice-cold 85:15 acetonitrile:water was then added. Samples were then vortexed, centrifuged, and then evaporated before being reconstituted in water and then analyzed by HPLC-MS/MS.

### Statistical analysis

Survival and immunohistochemical staining data were analyzed using GraphPad Prism software (GraphPad Software, La Jolla, CA). Survival data was presented as Kaplan-Meier curves, in which the statistical significance of the median survival times was assessed using a Log-rank test. Statistical significance in the immunohistochemical data was analyzed with a standard, unpaired t test. P values of less than 0.05 were considered significant.

## Results

### ROCK1 is overexpressed in pancreatic tumor tissues

**Fig 1:**
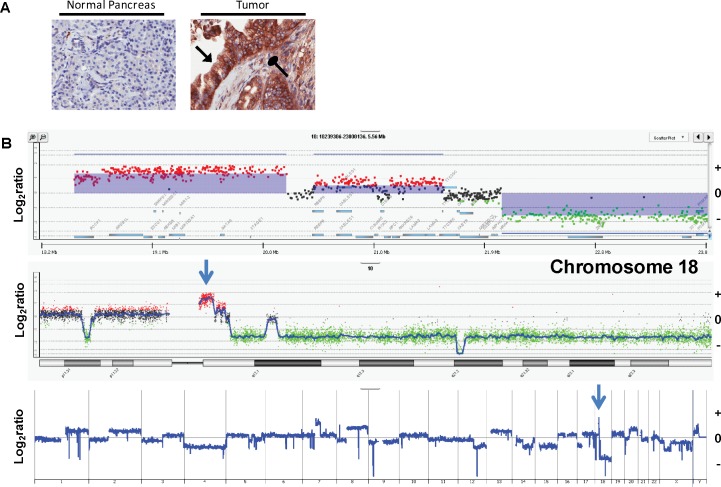
ROCK1 protein expression and gene amplification in PDAC tissues. A) Immunostaining of ROCK1 protein in a normal pancreas section (negative staining) and a PDAC tissue section (strong staining with a score of 3+). Black arrow: tumor cells; oval arrow: cancer associated fibroblasts. B) aCGH plots of the whole genome (bottom panel), chromosome 18 (middle panel) and the ROCK1 gene locus (top panel) from a representative patient with a focal (1.76Mb) 18q11.1 amplicon. Blue shaded areas denote aberrant copy number intervals defined by ADM2 step gram algorithm [31] with a log_2_ratio of +1.4 (p<0.001) for the ROCK1 containing amplicon.

For the current study, we analyzed the TMA of PDAC samples for expression of ROCK1 using IHC staining techniques. This analysis revealed that ROCK1 was highly expressed in the majority of our tumor tissue samples, both in the tumor epithelial cells and in the surrounding stromal cells (Fig 1A). We observed that 62.0% of tumor sections stained with an intensity of ≥2+. In separate, adjacent normal pancreatic sections (normal pancreas tissues adjacent to tumor), 40.4% stained with ≥2+ intensity. Only 33.3% of normal pancreas sections (from non-cancer patients) stained with a ≥2+ intensity (Table 1). The increased frequency of ≥2+ staining found in the tumor epithelial compartment was statistically significant (P = 0.005). Staining in tumor tissues was markedly non-uniform. Tumor epithelial cells demonstrated varied, but generally robust staining intensities. In contrast, CAF cells displayed punctate staining of varying intensity.

**Table 1:** Summary of immunohistochemical staining of ROCK1 in PDAC tissue microarrays

Tissue histology	Staining Intensity*					% Cases with ≥ 2+
	0	1+	2+	3+	N/E	
**Tumor (n = 133)**	8	38	42	33	12	62.0
**Adjacent normal pancreas (n = 59)**	7	24	17	4	7	40.4
**Normal pancreas (n = 12)**	4	4	3	1	0	33.3

* Staining intensities range from 0 to 3+ where 0 = negative, 1+ = weak, 2+ = moderate, 3+ = strong, and N/E = not evaluable due to lack of epithelial cells

Investigation of ROCK1 genomic aberrations identified a 1.76 Mb focal amplification (log_2_ratio 1.4) in chromosome 18q11.1 using array-comparative genomic hybridization (aCGH) analysis of flow sorted tumor samples. This region includes the ROCK1 gene locus (Fig 1B). This amplification was observed in four of thirty-four patient samples analyzed (12%).

### ROCK1 knockdown inhibits growth

**Fig 2:**
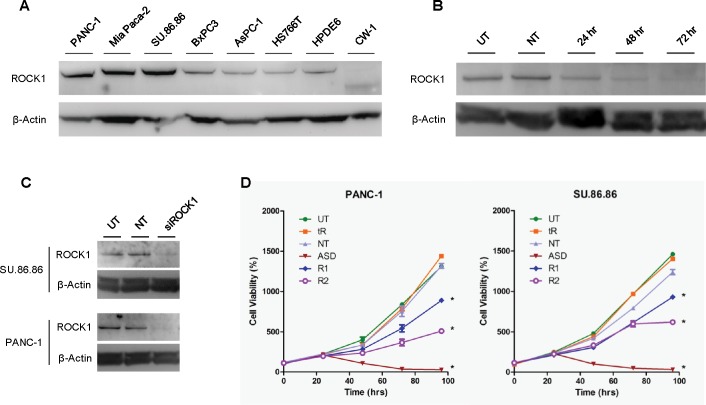
Knockdown of ROCK1 by siRNA in pancreatic cancer cell inhibits cell proliferation. A) ROCK1 was detected in pancreatic cancer cell lines (PANC-1, Mia PaCa-2, SU.86.86, BxPC3, AsPC-1, and HS766T), the immortalized normal pancreatic ductal epithelial cell line (HPDE6), and the cancer associated fibroblasts (CW-1) by Western blotting. B) Western blotting analysis of ROCK1 knockdown by siRNA over the course of 72 hours. (C) Western blotting analysis of ROCK1 knockdown by siRNA (72 hour treatment) in two cell lines, SU.86.86 and PANC-1. (D) Growth curves of pancreatic cancer cells (PANC-1 and SU.86.86) treated with siRNA to ROCK1. * P < 0.001 (compared to the untreated control). UT, untreated; tR, transfection reagent only; NT, non-targeting; ASD, cell death (positive) control; R1, ROCK1 siRNA1; R2, ROCK1 siRNA 2.

We first compared the expression of ROCK1 among multiple, established pancreatic cancer cell lines, as well as the immortalized normal pancreatic ductal epithelial cell line HPDE6 and the CAF cells (CW-1) isolated from a PDAC patient’s tumor. Western blotting analysis showed robust expression among the PANC-1, Mia PaCa-2, and SU.86.86 cell lines, and relatively low expression among the BxPC3, AsPC-1, and HS766T lines (Fig 2A). The HPDE6 cells also expressed low level of ROCK1. The CW-1 CAFs did not express significant levels of full length ROCK1, but rather appeared to express a ROCK1 antibody reactive protein of approximately 130 kDa, consistent with the molecular weight of a caspase-3 cleavage activated ROCK1 fragment [32]. To study the direct effects of ROCK1 expression, siRNA oligonucleotides were used to knock down its expression in the PANC-1 and SU.86.86 cell lines (Fig 2B and 2C). ROCK1 expression continued to decline over 72 hours following siRNA treatment (Fig 2B). ROCK1 siRNA treatment reduced the proliferation rate of PDAC cells. With time-course cell viability assays, we determined that decreased cell proliferation coincided with ROCK1 levels, where significant decreases in proliferation of PANC-1 and SU.86.86 cells was not observed until, or after, approximately 72 hours (Fig 2D). This effect was observed with multiple siRNAs and in two different cell lines (data not shown).

**Fig 3:**
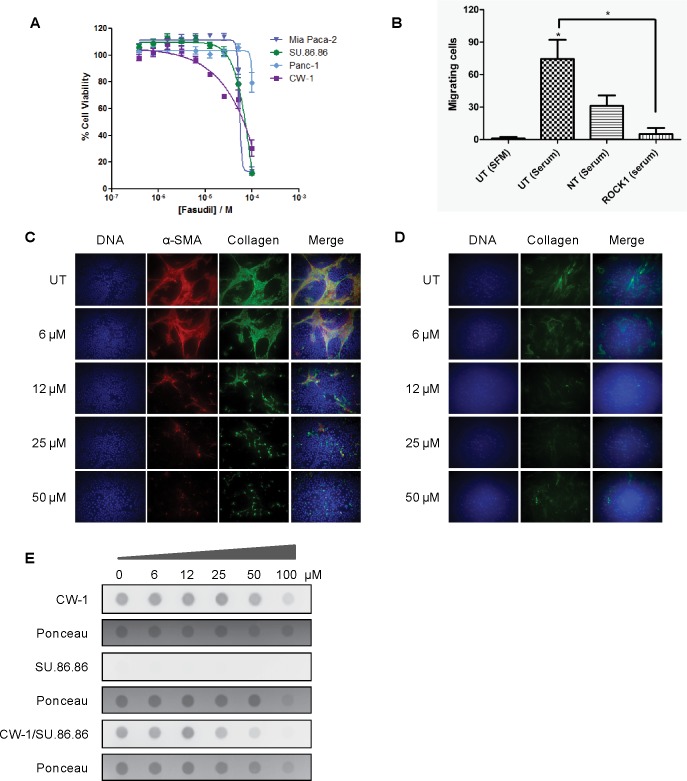
Effects of the ROCK1 inhibition on pancreatic cancer cells and cancer-associated fibroblasts. A) Fasudil dose response curves in pancreatic cancer cells treated for 72 hours. B) Tumor cell migration in ROCK1 siRNA treated cells. C) Fluorescence microscopic analysis of fasudil treated, co-cultured pancreatic cancer cells and cancer-associated fibroblasts. Cells were treated with fasudil for 48 hours and then were stained for α-SMA (red), Collagen I (green), and DNA (blue). D) Fluorescence microscopic analysis of fasudil treated CAFs. E) Fasudil treated, mono- and co-cultured pancreatic cancer cells and cancer-associated fibroblasts were harvested and analyzed by immunoblotting (dot blot) for Collagen I expression under non-denaturing conditions.

To compare siRNA knockdown results with enzymatic inhibition using small molecules, we tested the effects of the ROCK inhibitor, fasudil, on the cell proliferation of several cell lines using the SRB assay (Fig 3A). Fasudil is a well-described ROCK inhibitor that has shown clinical activity in treating cerebral vasospasm [33,34]. While prior reports have shown fasudil effects on normal rat stellate cells, to our knowledge, this is the first report of the effects of fasudil on human primary PDAC CAFs [35]. Consistent with our siRNA data, fasudil showed modest effect on the pancreatic cancer cell growth, and minimal effect on CAF cell growth, with IC_50_s in the 50–80 μM range. ROCK1 siRNA also significantly inhibited PANC-1 tumor cell migration (Fig 3B). To evaluate the effects of ROCK1 inhibition in a model more relevant to the *in vivo* environment, in which both tumor cells and CAFs are grown together, we treated co-cultured SU.86.86 cells with CW-1 cells, and tested the effects of fasudil on phenotypic properties of the cultured cells. Fasudil treatment reduced the phosphorylation of MYPT1, a ROCK1 target protein (S1 Fig), which is consistent to prior reports [35]. Using immunofluorescence microscopy (Fig 3C), we detected α-SMA and Collagen I expression in co-cultured cells treated with a range of fasudil concentrations for 72 hours. DNA staining reveals that significant numbers of α-SMA-negative cells (tumor cells) remain on the slide with even high concentration of fasudil. α-SMA-positivity (CAFs) was largely lost following fasudil treatment in the context of co-culture, despite showing similar IC_50_ values in monoculture with fasudil treatment. Monocultured CAFs treated with fasudil also show some loss of α-SMA positivity, suggesting some reversion of CAF activation may be occurring (Fig 3D). Importantly, this reduction in CAF cell proliferation or activation coincided with reduction in total Collagen I expression, whether assessed qualitatively by microscopy, or semi-quantitatively by dot-blotting under non-denaturing conditions (Fig 3E).

**Fig 4:**
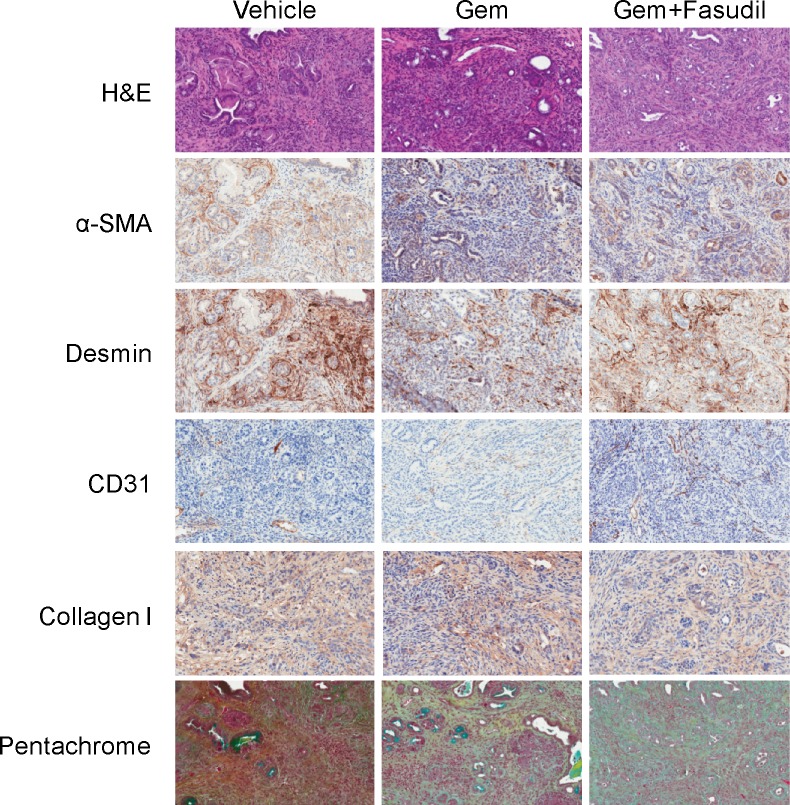
Effects of fasudil treatment on tumor stroma in KPC mice. A) Pancreatic tumor tissues from vehicle, gemcitabine, or combination of gemcitabine plus fasudil treated KPC mice were harvested and stained for various stromal markers. Representative images are shown of H&E staining, α-SMA, Desmin, CD31, Collagen I, and Movat's pentachrome staining.

To determine if these reductions in collagen expression observed *in vitro* would also translate to reductions *in vivo*, we used a genetically engineered mouse (GEM) model for PDAC (the KPC mouse model) to evaluate the effects of fasudil and gemcitabine treatment *in vivo*. As shown in Fig 4, significant changes in α-SMA and Desmin expression were not observed. However, significant reduction in collagens was observed with the combination treatment of gemcitabine plus fasudil in tissues stained with a Collagen I antibody and a pentachrome stain. The pentachrome stain highlights collagens with a distinct and measurable yellow coloring. The yellow staining was markedly diminished in the gemcitabine plus fasudil treated mouse tissues (Fig 4).

### ROCK1 inhibition does not enhance tumor cell metastasis

**Fig 5:**
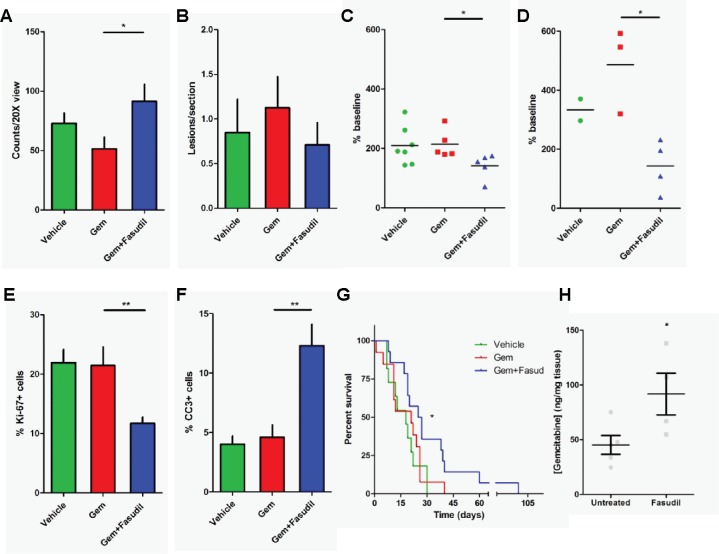
Effects of gemcitabine and fasudil treatment in the KPC model for PDAC. A) Pancreatic tissues harvested and stained for CD31 were analyzed for positive staining per 20x field of view. B) Mouse liver sections were cut at multiple depths to assess the presence or absence of metastatic lesions in vehicle, gemcitabine, and gemcitabine plus fasudil treated tissues. Fasudil also enhanced the tumor growth inhibitory activity of gemcitabine in KPC mice. Three-dimensional volume measurements were acquired by ultrasonography of tumor bearing KPC mice before and after one (C) or two (D) 12-day treatment cycles. Percentage change is shown from baseline. IHC analysis of the proliferation marker Ki67 (E) and apoptosis marker cleaved-caspase 3 (CC3) (F) in epithelial tumor cells are also shown for mice treated in the various treatment cohorts. Fasudil enhanced survival of tumor bearing KPC mice. G) Kaplan-Meier curves of KPC mice treated with vehicle, gemcitabine, or the combination of gemcitabine plus fasudil. The combination treatment yields a significant improvement in overall survival compared to gemcitabine only group (Log-rank P value = 0.038). H) Pancreatic tissue from mice treated for three days with either vehicle or fasudil prior to a gemcitabine injection were analyzed for gemcitabine monophosphate concentrations in the tumor tissues. * P < 0.05. ** P < 0.01.

Due to concern that therapeutic alteration of the tumor microenvironment, including reducing collagen content, might disrupt tissue integrity and increase tumor cell propensity for metastasis, we looked for signs of this outcome in mouse tissues with two endpoints: mean vessel density in the pancreas, and incidence of liver micro-metastases (Fig 5). Changes in mean vessel density can arise due to multiple causes, including compression of stromal tissues (e.g., inhibition of CAF cell proliferation) or angiogenesis. While significant changes in CD31 staining intensity were not seen with treatment (Fig 4), a statistically significant increase in CD31 positive cell counts was observed in the combination treatment, relative to gemcitabine alone (Fig 5A, P = 0.035). To determine if the drug treatment resulted in increased incidence of metastasis to the liver, FFPE fixed liver sections from treated mice were inspected and counted for incidence of tumor nests (Fig 5B). There was no statistically significant difference between the combination treatment, and that of either vehicle or gemcitabine treated alone (P = 0.618 and 0.755, respectively).

### ROCK1 inhibition potentiates anti-tumor activity in KPC mice

To determine if this combination treatment translated to measureable differences in tumor growth *in vivo*, we followed tumor volume changes pre- and post-treatment using three-dimensional ultrasonography (Fig 5C and 5D). Relative percent change is displayed after one (Fig 5C) or two (Fig 5D) 12-day cycles of vehicle, gemcitabine, or gemcitabine plus fasudil. While small, significant reductions in overall tumor volume growth were seen after the first 12-day cycle, more significant growth reductions were observed with a second 12-day treatment cycle, in the combination treatment group versus vehicle or gemcitabine alone. Of note, fewer mice were lost to disease burden in the combination treated group versus the vehicle or gemcitabine treated groups.

We next analyzed tissue from treated mice, to assess cellular changes occurring in response to treatment. We looked at two markers of drug activity, Ki67 for cell proliferation and cleaved caspase-3 (CC3) for cell apoptosis. After a 12-day cycle, significant decreases in proliferation (as assessed by Ki67, Fig 5E) were observed relative to vehicle or gemcitabine treatment. Increases in apoptotic cell counts (CC3-positive, Fig 5F) were observed in the tissues of these treated mice.

### ROCK1 inhibition correlates with better survival and drug delivery in the KPC model

We next performed an intervention survival study in the KPC mice. The median survival of vehicle and gemcitabine treated mice was 18 and 21 days, respectively. The median survival for the combination treatment was statistically greater than gemcitabine alone, at 26 days (P = 0.038, Log-rank test) (Fig 5G). The hazard ratio for combination treatment was 2.16. To assess whether histological changes in Ki67 and CC3 expression, as well as changes in overall survival, might be associated with gemcitabine activity, we looked at gemcitabine concentrations within tumors of treated mice. Mean gemcitabine monophosphate (dFdCMP) concentrations in vehicle treated mice were 45.3 ng/ mg tissue. In fasudil treated mice, mean gemcitabine monophosphate (dFdCMP) concentrations reached 91.8 ng/mg tissue (a 102.6% increase). While one explanation for this difference might include altered serum levels of gemcitabine, our data demonstrates that fasudil treatment increased the concentration of gemcitabine into the tumor (Fig 5H). Additional studies will be needed to delineate tumor perfusion of gemcitabine and serum accumulation.

## Discussion

In the current study, we investigated the hypothesis that ROCK1 plays a role in physiological chemoresistance and mediates the reduced efficacy of chemotherapeutics in pancreatic cancer. Pancreatic tumor masses are poorly perfused, with desmoplasia resulting in more than a 65% reduction in tissue perfusion, which can translate to a nearly 60% reduction in tumor gemcitabine uptake in the KPC mouse model [15,26]. By immunohistochemical (IHC) analysis, we observed an increased expression of ROCK1 protein in the majority of PDAC patients, with increased expression in both the tumor epithelial compartment and stromal cells, relative to adjacent normal pancreas, or normal pancreas tissues. This phenomenon could be explained, at least in part, by a focal gene amplification though this was observed in only a small subset of our PDAC patients.

Consistent with prior reports, our study shows that ROCK1 is important for not only migratory movement of tumor cells, but also ECM deposition in CAFs. In the KPC model, treatment with a ROCK1 inhibitor (fasudil), in combination with gemcitabine, inhibits collagen deposition in tumors. This effect coincides with increases in CD31 positivity, an indirect measure of tissue vascularity, but does not correlate with metastasis. Though CD31 increases coincide with combination treatment, this observation is likely a consequence of tumor debulking, and reductions in ECM proteins. Fasudil does, however, inhibit tumor growth significantly by both decreasing proliferation of tumors and increasing apoptosis. Fasudil, in combination with gemcitabine, contributes to the improvement in survival of the KPC mouse by five days, presumably by increasing the effective tumor concentrations of gemcitabine. While prior studies report a different median survival of vehicle treated KPC mice (10.5 days), our studies were performed with tumors of slightly smaller average size (100 mm^3^), and may reflect a less advanced disease state [28].

Increased ROCK1 expression has been observed in a number of pathological conditions, including cancer [17,18]. Multiple studies have documented the role of ROCK in fibrosis [36,37]. Indeed, somatic mutations associated with increased catalytic activity in ROCK1 have been previously described in gastric carcinoma and melanoma [16]. Though endothelial nitric oxide synthase (eNOS) and the PI3K/Akt pathway appear to be implicated in ROCK signaling in cardiac models [37], ROCK1 in pancreatic cancer or CAFs specifically is less well understood. Importantly, reports have shown that ROCK1 inhibition can reduce pancreatic CAF function and their secretion of ECM proteins [35]. However, the potential for ROCK1 inhibition to enhance chemotherapeutic regimens has not been investigated in a proper model which recapitulates the pancreatic tumor microenvironment. Although inhibition of ECM synthesis and deposition in pancreatic stellate cells has been reported previously with ROCK inhibitors [35], we add to these findings by demonstrating that the anti-CAF effects are also observed *in vivo*, and that these anti-collagen effects coincide with increased anti-tumor efficacy. While we cannot exclude that fasudil-mediated anti-tumor effects are independent of gemcitabine, our data supports the hypothesis that stromal deposition of collagen and other ECM proteins impedes drug delivery. Further study into more potent ROCK1 inhibitors may give insight into the cell type-specific activity by which ROCK1 mediates tumor progression *in vivo*. The current report is consistent with the notion that stromal inhibition can enhance therapeutic efficacy of gemcitabine by mediating decreased ECM deposition and enhanced gemcitabine uptake.

There is growing debate over the clinical utility of stromal targeting in pancreatic cancer. The prevailing theory of recent years has been that the tumor microenvironment promotes tumor growth [38–40]. Multiple studies have demonstrated the involvement of tumor promoting ECM-integrin α_5_β_1_ interactions, tumor enhancing hyaluronan-CD44 interactions, and CAF-mediated TGFβ secretion in tumor progression [41–45]. On the other hand, two recent studies have called that hypothesis into question, suggesting that the stromal cells act instead to restrain the growing tumor [46,47]. Certainly, the clinical failure of IPI-926, a sonic hedgehog inhibitor which failed in clinical trial despite promising preclinical results in the KPC mice (clinicaltrials.gov, NCT01130142), suggests we do not fully appreciate the problem. The stromal targeting theory is again facing scrutiny with a recombinant human hyaluronidase undergoing investigation in a phase III trial in stage IV PDAC (clinicaltrials.gov, NCT02715804) [48,49].

Özdemir and colleagues reported in a comprehensive set of experiments, that α-SMA positive cell (the CAF cell population) ablation resulted in a statistically significant decrease of 3–4 days in overall survival in the PKT mouse model for pancreatic cancer (P = 0.0143) [46]. This runs counter to the notion that inhibition of CAF function and ECM deposition will reduce tumor growth. It should be noted, however, that Özdemir remarks that in the PKT depleted model there is a significantly increased incidence of pulmonary emboli, and TUNEL positivity, even in the absence of gemcitabine treatment, both of which may be confounding factors in their survival study not delineated in a multivariate analysis.

We believe one aspect could explain the discrepancy between their findings and the current study, namely, that ROCK inhibition acts in both a tumor cell autonomous and non-cell autonomous manner. Indeed, ROCK1 may be playing an active role in both tumor and stromal cells. Some researchers have recently voiced concerns over the possible increase in tumor metastasis with stromal targeting. Here we did not find this to be the case with ROCK inhibition, nor has it been true in other studies of stromal targeting [40]. We and others have observed that fasudil decreases tumor cell migration *in vitro*. This effect may play significantly in its efficacy in the current study. We have observed that though collagen is decreased in the gemcitabine plus fasudil treated tissues, α-SMA positivity is not altogether eliminated. Further investigation of the relationship between α-SMA positivity and collagen deposition is needed in PDAC.

We are of the opinion that these two phenomena—that the tumor microenvironment both promotes tumor growth and restrains advanced progression—are not diametrically opposed, especially when considering therapeutic outcomes. ECM proteins, such as Collagens I, III, and IV, are secreted at high levels in PDAC. Tumor collagen content, specifically, has been shown to maintain an inverse relationship to that of macromolecule penetration. Collagens I, III, and IV have been shown to bind small molecules and impair their diffusion through tissue by more than 10-fold in some models [11]. ECM deposition also contributes to decreased drug penetration by mediating increases in interstitial fluid pressures (IFP) and solid stress in tumor tissues [40,50,51]. Proper maintenance of IFP and intravascular pressure (IVP) is required for the perfusion of nutrients as well as the systemic delivery of chemotherapeutic agents. Recently, it was demonstrated that the pancreatic IFP in the genetically engineered KPC mouse model increased from an average of 10.4 mm Hg in normal tissues to upwards of 99 mm Hg in the diseased pancreas [49]. This stands in strong contrast to the average IVP of 40–80 mm Hg in tumor tissues. When IFP surpasses IVP, normal flow of various solutes, including chemotherapeutics, is hindered. This striking difference is thought to account for the poor efficacy of therapeutics in PDAC [49].

Rath et al recently report related findings on ROCK1 and 2 in PDAC [52]. The current study adds to their findings, reporting on the amplification of human ROCK1 gene in a subset of PDAC patients, as well as additional mechanistic insights on the consequence of inhibition of collagen remodeling on vasculature and drug (gemcitabine) uptake.

Taken together, our results demonstrate that ROCK1 inhibition can reduce the functional impact of the activated CAF population by reducing collagen deposition both *in vitro* and *in vivo*. This coincides with increased survival of the KPC mouse, and enhanced concentrations of intratumoral gemcitabine. Based on our findings, we feel that ROCK1 inhibition may constitute an alternative approach for inhibition of the stromal protection provided in PDAC. We are confident that through this and other ongoing studies, treatment regimens incorporating stroma-targeted agents will make an impact on the survival of patients with advanced pancreatic cancer.

## Supporting information

S1 Fig Effect of Fasudil treatment on the phosphorylation of ROCK1 substrate MYPT1. SU86.86 cells were treated with Fasudil for 24 hours at the concentration indicated. Cells were harvested and Western blotting analysis of 30 μg of total protein lysate was carried out using the same procedures as described in the Materials and Methods section. The primary antibodies used include anti-MYPT1 from Cell Signaling (Cat #Cat 8574) at 1:750 dilution; anti-pMYPT1 (Thr696) from Cell Signaling (Cat #5163) at 1:750 dilution; and anti-β-actin from Sigma-Aldrich (Cat #A5316) at 1:2000 dilution.
(EPS)
